# Integrin α3 mutation junctional epidermolysis bullosa presenting with hypotrichosis and lacrimal obstruction

**DOI:** 10.1016/j.jdcr.2023.10.007

**Published:** 2023-10-30

**Authors:** Hind Alshihry, Shahad D. AlGhamdy, Amal Alhashem, Hind M. Almohanna

**Affiliations:** a Department of Dermatology, Prince Sultan Military Medical City, Riyadh, Saudi Arabia; b Department of Dermatology, King Fahad Medical City, Riyadh, Saudi Arabia; c Department of Pediatrics, Prince Sultan Military Medical City, Riyadh, Saudi Arabia

**Keywords:** epidermolysis bullosa, ILNEB syndrome, nephrotic syndrome

## Background

Integrin α3β1 is a transmembrane protein that plays a fundamental role in the basement membrane function in the epidermis, kidney, and lung, and its loss causes severe dysfunction of these organs. Junctional epidermolysis bullosa (EB) with nephrotic syndrome and interstitial lung disease (ILNEB syndrome) is a rare variant of EB caused by biallelic mutations in the integrin α3 (ITGA3) gene.^1^ Eight patients with ILNEB syndrome were reported who presented in the first few months of life, and the majority died during the first years of life due to severe pulmonary distress or renal failure.^1^, ^2^, ^3^, ^4^, ^5^, ^6^ Nine patients were reported with a milder phenotype with or without renal or respiratory manifestation who lived longer than those who previously reported.^7^, ^8^, ^9^, ^10^, ^11^, ^12^, ^13^ We present a variant of ILNEB syndrome with prolonged survival, past early childhood and without renal or respiratory symptoms.

## Case

**Fig 1:**
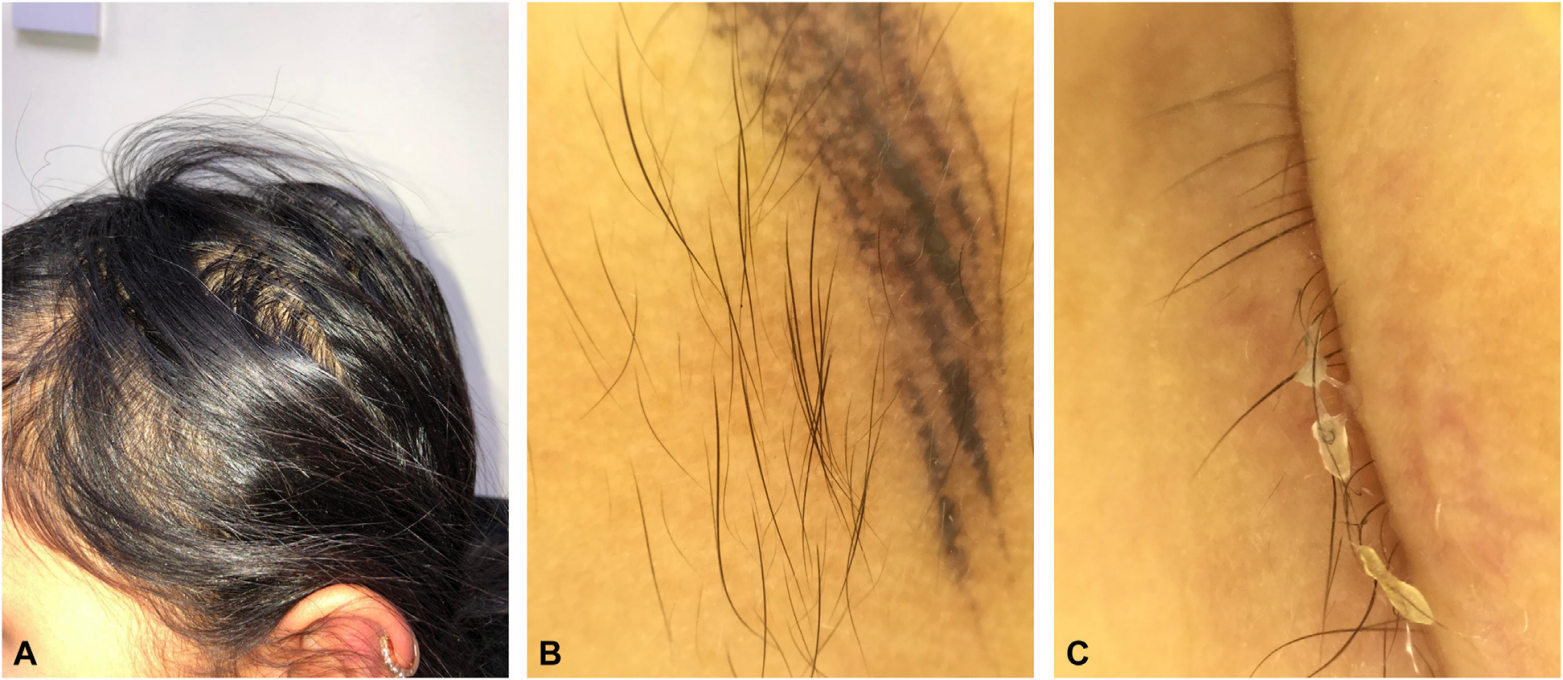
**A–C,** Hypotrichosis in the 15-year-old patient. Similar presentation is found in the 13-year-old patient. **A**, Diffuse decrease in the hair density over the scalp. **B**, Dermatoscopy shows spars thin hair of the left eyebrow with eyebrows tattoo. **C**, Thin spars eyelashes.

**Fig 2:**
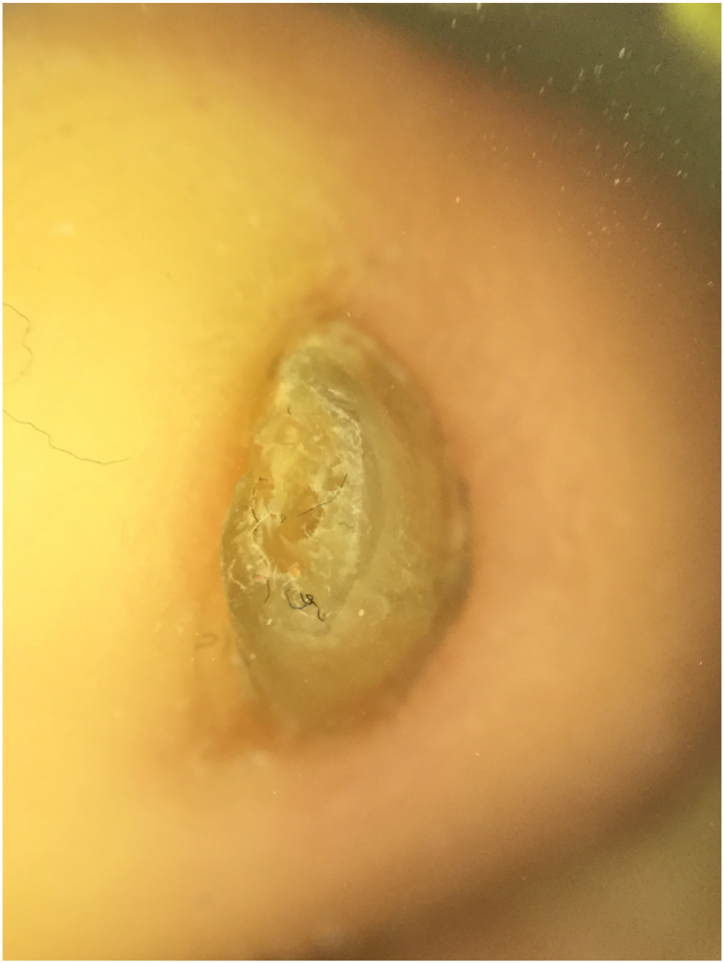
Dermatoscopy showed thick curved nail plate of the right side of the big toenail in the 15-year-old girl.

**Fig 3:**
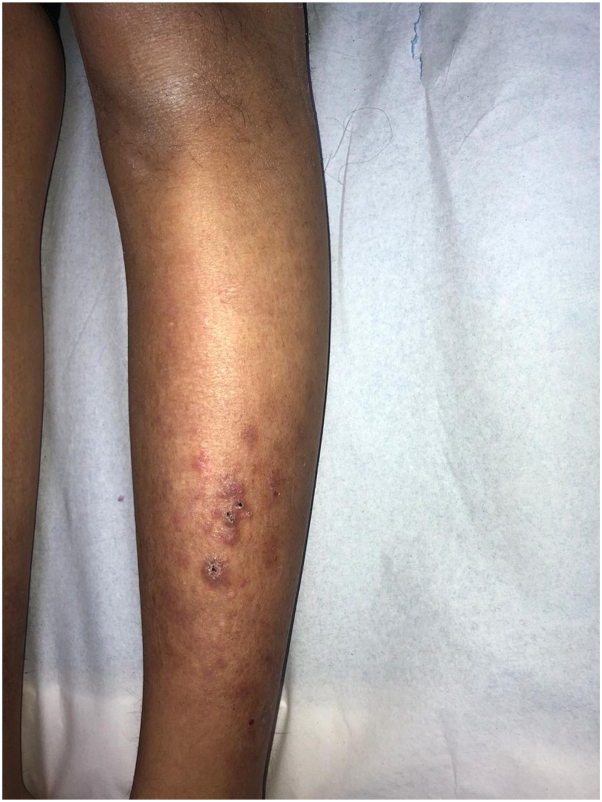
Hyperkeratotic papules on bilateral shins in 15-year-old girl.

**Fig 4:**
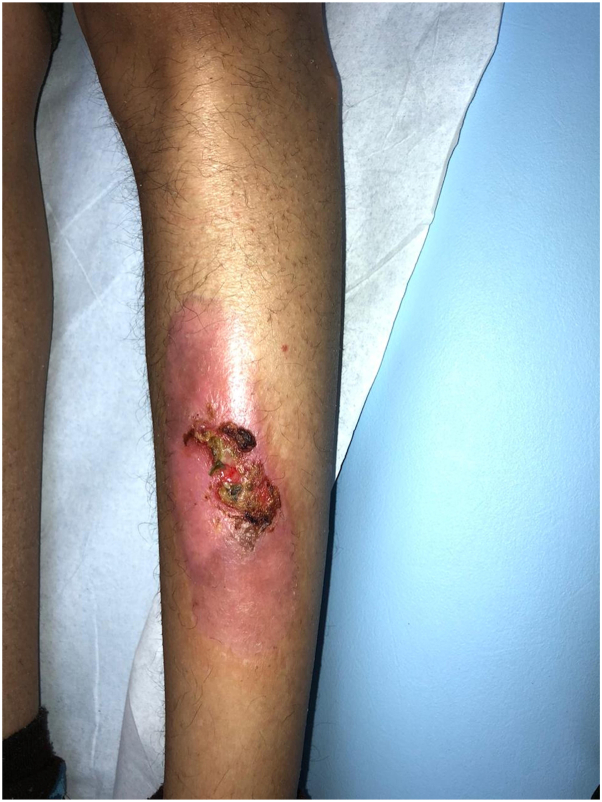
Right pretibial erythematous atrophic patch with intact blister in 13-year-old boy.

We report 3 siblings of 15-year-old female, 13-year-old male, and a 2-year-old female, born to consanguineous healthy parents. They were well until the age of few months, when they started to have gradual decrease in the density of scalp and body hair, as well as loss of eyebrows and eyelashes (Fig 1). The 15-year-old female and the 13-year-old male developed skin fragility, nail dystrophy (Fig 2), and lacrimal duct obstruction at the age of 2 years, without respiratory or urinary symptoms. The 2-year-old patient presented with only hypotrichosis. The skin fragility in the eldest was in the form of itchy erythematous papules and plaques that appeared over both upper and lower extremities (Fig 3). Her brother presented with a blister on his right shin following minor trauma, that healed, leaving an erythematous atrophic patches with central erosion and crust (Fig 4). The parents denied systemic symptoms, respiratory symptoms, sweating problems, heat intolerance, dentitions, or deafness. Targeted DNA analysis for the 3 siblings using exome sequencing detected a homozygous variant of missense mutation (c.821G>A), causing an arginine to glutamine amino acid substitution at position 274 (p.Arg274Gln) in the ITGA3 subunit.

Blood creatinine levels and findings of urinalysis, renal ultrasound, pulmonary function test, chest X-rays, and high-resolution computed tomography scans were normal. All patients are following with a pulmonologist and a nephrologist for observation as well as dermatologist.

## Discussion

ITGA3 deficiency leads to ILNEB syndrome, it is an autosomal recessive disorder first described in 2012.^1^ The clinical manifestations of ITGA3 deficiency consist of interstitial lung disease, renal anomalies (including nephrotic syndrome, focal segmental glomerulosclerosis, bilateral renal cysts, and a spectrum of congenital anomalies of the kidneys and urinary tracts were reported), and skin fragility. The skin involvement was variable, including blisters, erosions, itchy excoriated papules, erythema, skin atrophy, scarring, dyspigmentation, poikiloderma, mucosal erosion, nail dystrophies, or sparse hair.^11^ Seventeen patients with ITGA3 mutations have been reported.^1^, ^2^, ^3^, ^4^, ^5^, ^6^, ^7^, ^8^, ^9^, ^10^, ^11^, ^12^, ^13^ Out of these, 8 experienced renal and respiratory symptoms either at birth or within the first few months of life.^1^, ^2^, ^3^, ^4^, ^5^, ^6^ Unfortunately, most of the patients who exhibited systemic symptoms early in life passed away within a few months of birth or during early childhood, with one exception. This particular case, reported by Okamoto et al,^6^ involved a patient who underwent a renal transplant at the age of 4 years. At the time of publication, the patient had been followed up for 8 months and had exhibited good tolerance to the transplantation.^6^ The remaining 9 initially showed diverse dermatological symptoms, followed by renal and/or respiratory symptoms that appeared later and were less severe than the earlier cases.^7^, ^8^, ^9^, ^10^, ^11^, ^12^, ^13^ They also had a longer lifespan compared with those who experienced early systemic symptoms. Colombo et al^11^ reported 2 siblings who had compound heterozygous ITGA3 mutations (c821G>A, P.Arg274Gln) and (c.373G>A, p.G125R) and it was suggested that the (c821G>A, P.Arg274Gln) mutation was responsible for their survival and lack of renal involvement.^11^ Also, Cohen-Barak et al^12^ reported patient with c821G>A mutation but in homozygous, and experienced skin fragility and lacrimal gland obstruction without any other systemic symptoms, which is similar to our cases.^12^ Notably, the missense mutation (c.821G>A: Arg274Gln) may be responsible for the relatively mild ILNEB symptoms observed in these individuals, as suggested by Colombo et al.^11^^,^^12^

Lacrimal duct obstruction with tearing early in life were seen in 5 of the patients that were reported previously and 2 of our cases, which widen the phenotypic spectrum and should increase the suspicion of ILNEB syndrome if associated with erosions of the skin and sparse hair.^4^^,^^7^^,^^11^, ^12^, ^13^

Considering the broad phenotype presentations of ITGA3 mutation junctional EB, hypotrichosis as the only presenting feature in a patient should raise the suspicion of junctional EB when formulating the differential diagnosis, and trigger further molecular testing. Patients with integrin 3 mutation need regular follow-up for pulmonary and renal involvement, even if they lack symptoms early in life. This will allow an early diagnosis of the disease to initiate appropriate management of the complications and genetic counseling.

## Conclusion

We report 3 siblings presenting with hypotrichosis with or without lacrimal gland obstruction, skin erosions or blistering, and nail dystrophy as showing signs of a variant of ILNEB syndrome with homozygous mutations in the ITGA3 gene and lacking renal and lung involvement. We should keep in mind the phenotypic diversity of ILNEB when constructing the differential diagnosis and making treatment decisions.

## Conflicts of interest

None disclosed.

## References

[1] Has C., Spartà G., Kiritsi D. (2012). Integrin α3 mutations with kidney, lung, and skin disease. N Engl J Med.

[2] Nicolaou N., Margadant C., Kevelam S.H. (2012). Gain of glycosylation in integrin α3 causes lung disease and nephrotic syndrome. J Clin Invest.

[3] Yalcin E.G., He Y., Orhan D., Pazzagli C., Emiralioglu N., Has C. (2015). Crucial role of posttranslational modifications of integrin α3 in interstitial lung disease and nephrotic syndrome. Hum Mol Genet.

[4] He Y., Balasubramanian M., Humphreys N. (2016). Intronic ITGA3 mutation impacts splicing regulation and causes interstitial lung disease, nephrotic syndrome, and epidermolysis bullosa. J Invest Dermatol.

[5] Sondermann W., Büscher R., Forster H. (2021). Skin fragility, renal malformation and interstitial lung disease due to compound heterozygous ITGA3 mutations. J Deutsche Derma Gesell.

[6] Okamoto T., Nakamura A., Hayashi A. (2021). Successful kidney transplantation in a patient with neonatal-onset ILNEB. Pediatr Transplant.

[7] Tarur S.U., Srinivasan S., Seeralar A. (2020). Delayed presentation of respiratory symptoms and prolonged survival in homozygous a3 integrin deficiency. Indian Pediatr.

[8] Alstrup M., Marks S.D., Ek J. (2021). First patient with ILNEB syndrome due to pathogenic variants in ITGA3 surviving to adulthood. Eur J Med Genet.

[9] Liu Y., Yue Z., Wang H. (2021). A novel ITGA3 homozygous splice mutation in an ILNEB syndrome child with slow progression. Clin Chim Acta.

[10] Atmis B., Cevizli D., Cagli C. (2023). Nephrotic syndrome, skin involvement, and chronic lung disease: answers. Pediatr Nephrol.

[11] Colombo E.A., Spaccini L., Volpi L. (2016). Viable phenotype of ILNEB syndrome without nephrotic impairment in siblings heterozygous for unreported integrin alpha3 mutations. Orphanet J Rare Dis.

[12] Cohen-Barak E., Danial-Farran N., Khayat M. (2019). A nonjunctional, nonsyndromic case of junctional epidermolysis bullosa with renal and respiratory involvement. JAMA Dermatol.

[13] Kinyó Á., Kovács A.L., Degrell P. (2021). Homozygous ITGA3 missense mutation in adults in a family with syndromic epidermolysis bullosa (ILNEB) without pulmonary involvement. J Invest Dermatol.

